# Etiology and Treatment of Talipes, Varus and Vulgus, Congenital and Acquired

**Published:** 1888-03

**Authors:** C. McReynolds

**Affiliations:** Fort Smith, Arkansas


### ETIOLOGY AND TREATMENT OF TALIPES, VARUS AND VULGUS, CONGENITAL AND ACQUIRED.

*By C. McReynolds, M. D., Fort Smith, Ark.*

For Daniel's Texas Medical Journal.

THE progress made in orthopædic surgery within the past decade surpasses by far that made in a century before, both as regards clinical study and experimental investigation. This very important department of surgery has been, seemingly, in a state of "innocuous desuetude," whilst other departments have been studied with unparalleled zeal and industry. Surgeons have been, heretofore, apparently powerless, and viewed the correction of these deformities as foreign to their art; the crippled and deformed lived and moved in their midst, bearing on their countenances the impress of mortified pride, living reproaches to the claims of progressive surgery. It is now, however, gratifying to note that within a comparatively brief space of time, mechanical surgery has gained and holds a very enviable position.

Nothing original shall be attempted in this paper and my only excuse for offering an article to the already engorged medical press is that having served an apprenticeship in an orthopædic institution, I hope to be able to present a few practical facts the result of personal work and observation, facts which I trust may be of some service at least to a few.

Before proceeding to my subject I take it, that orthopædic surgery properly includes any deformity that requires for its correction and restoration mechanical support; a fracture, an osteitis belong to orthopædic work as certainly as do club feet and spinal caries.

Varus in combination with equinus is perhaps the most frequently met deformity of the foot; in fact I do not remember ever seeing an uncomplicated case of varus; vulgus, a condition involving the tibial group of muscles, is directly the reverse of varus, and forms comparatively a very small per cent. of the deformities of the foot.

The etiology of varus and vulgus, and indeed all forms of talipes is still very obscure; like all questions in doubt, everyone interested has assumed the onus of projecting an opinion, some with, and some without reason; 'twould be useless to discuss in detail these various opinions; I will therefore only mention a few: A diminution of the liquor amnii, causing an undue amount of pressure by the uterus, a fright, a fall, tight lacing, an unaccountable lusus naturæ, have with many other reasons been assigned. The most modern and apparently the most rational theory is that which attributes it to a paralysis—a paralysis, the result either of prolonged uterine pressure, holding the extremities in an abnormal position, or the result of a cord lesion or the sequence of some of the blood poisoning diseases affecting the fœtus in utero.

It was formerly thought and taught that the muscles on the side to which the foot was drawn were the ones at fault in producing the deformity, and this error as to causation often led to very gross procedures in treatment. Anyone may convince himself of the paralytic nature of the muscles, even in the newly-born, flabby and atrophied condition, also the facility with which the shortened tissues yield to traction, proving they have not undergone structural changes, that they are contracted and not contractured, that there is a lack of normal contractility in the opposing group.

Acquired talipes in the majority of cases is the result of infantile paralysis or of scarlatina, diptheria, etc., often, however, paralysis does not enter the case, "stone bruises," direct injuries inducing a spastic condition of the muscles frequently produce these deformities; "a spastic is often added to a paralytic condition." Spastic contractions have, until recently, been considered in all forms of talipes the sole pathological condition; they are now considered the exception, paralysis the rule. By spastic conditions I mean a spasmodic condition of the muscles arising from an irritation or inflammation, either of bone, muscle or fasciæ. This irritation and consequent spastic condition is often the result of a malposition.

The seat of varus and vulgus has been and is still an agitated question; it would seem, at first sight, that the deformity existed at the ankle joint, but upon a careful study of anatomy of this joint it is found that the projecting malleoli prevent any lateral movement whatever, that flexion and extension are the only movements of which it is capable, that it is purely a ginglymoid joint, an opinion, however, in which all do not concur. Another and more potent reason for these deformities is furnished by the fact, that of the twelve muscles that move the foot, three are attached to the os calcis and nine anterior to the medio tarsal junction, a joint that is formed posteriorly by the os calcis and astragalus anteriorly by the scaphoid and cuboid bones, and which admits of flexion, extension and an unquestioned degree of lateral movement. The natural deduction from such an anatomical arrangement is that since the muscles involved in varus and vulgus have their insertion anterior to this joint, traction made would inevitably produce a deformity at the point of least resistance, namely: the *medio-tarsal junction.*

Treatment: The treatment of congenital talipes should begin at birth, and in the acquired form there should be no unnecessary delay; the foot should at once be brought as nearly into normal position as possible, without inducing inflammation; do not wait until the child grows "a little stronger," as was once the custom. Active measures should also be made to increase the nutrition of the parts; all endeavors, however, on the part of the surgeon will prove fruitless, unless he has the hearty co-operation of parents or nurses; indeed, this is a *sine qua non* in the treatment of deformities, and there is no therapeutic agent that will, *per se*, accomplish our purpose; a multiplicity of resources are needed, and even then we are often put to our trumps to choose the best for each individual case.

Among the therapeutic agents, electricity stands first, massage; kneading, hypodermic injections of strychnia, strychnia ointment are all indispensible agents locally; constitutional treatment should not be disregarded.

I shall not attempt to describe the various intricate instruments that have been invented for the relief of those deformities, but shall only mention in this paper those methods most commonly adopted and most easy of application. Numberless modifications of the Scarpa shoe are in use, and many retard rather than hasten recovery. It may be said that the following is rapidly becoming an anxiom in orthopædic surgery, i. e. the simplest apparatus both in construction and adaptability is invariably the most satisfactory.

Motion is the normal state of muscle, to admit of activity; to imitate, as near as possible, natural movements, should be the dominant idea in treatment, and any apparatus applied that excludes free motion as physiologically wrong; moreover, no instrument should be applied that could not be easily removed for the purpose of electricy, massage, etc. In infants I have seen rapid progress from the use of the straight splints.

Case J. F. Aet., six weeks, presented at the dispensary (for ruptured and crippled) with double equine varus, Electricity and strychnia ointment were ordered, straight splints, three quarters of an ich wide and in length extending from head of fibula to the sole of the foot, the foot having been brought into normal position; the splints being nicely padded, were applied and held in position by the cotton roller. These were worn constantly for six months. At the expiration of this time a more permanent apparatus was usually applied, but in this case the feet had nearly assumed a normal state. Electricity and massage were continued and complete recovery finally ensued. The chief source of good is probably the fact that the splints are easily displaced, and consequently require frequent manipulation of the foot to retain in position.

The most adjustable and satisfactory splints are those made from sole leather.

Case A. F., Aet. eighteen months. Varo-equinus. A piece of leather board, enough to encircle about two-thirds of the circumference of the limb, having been thoroughly immersed in cold water, was first moulded to the foot, then to the leg, and retained in position by roller, until hardened; it was then removed, nicely padded and reapplied; removed daily for massage, shampooing etc.; electricity applied every other day, and at the end of seven months the foot, without support, retained a normal position and muscles had regained almost normal contractibility. Electricity was continued a few months longer to add further strength to the muscles, when patient was discharged.

I have seen happy results from the use of adhesive plaster. A strip is cut three to five inches in width and long enough to go nearly around the foot and about half way up the thigh; beginning on the dorsum of the foot, the plaster should be carried around the sole in the direction in which the foot is to be drawn carry the plaster as far as head of fibula, apply over this the roller, then reversing the end of the plaster, reapply the roller. The plaster should never encircle the foot for fear of retarding the circulation; the most potent objection to the plaster are the excorrations which invariably result when worn for any great length of time.

The above methods of procedure are very simple and require no special skill, and any practitioner who is willing to exercise more than ordinary patience and perseverance, may do much by these means toward the permanent restoration of these deformaties.

The following requires a special skill for its application. Artificial muscles, as a permanent tractile force, are often needed to overcome the rigidity of the muscles, and they are used with encouraging results. A triangular strip of adhesive plaster is cut into a numbar of strips, all converging to the apex; at the apex a wire hook is secured; the plaster is then applied to the foot, the hook located at a point that will represent the insertion of the affected muscle. To represent the origin a tin plate is cut, in length about two-thirds that of the tibia, and in width about one quarter of the circumference of the limb; about one inch from upper end of tin strip secure an eye, the tin is held partly by two adhesive strips long enough to encircle the limb, and through the centre of each, two slits are cut, through which the tin is passed. Another strip of plaster, twice the length of the tin and somewhat wider, is applied to the limb on the side where traction is to be made; on this place the tin strip, the upper end being level with the plaster, pass the upper strips around the limb, reverse on the tin the long vertical strip, cutting an opening for the eye, and over all pass a nicely adjusted roller which retains everything in proper position. The artificial limbs are represented by india rubber tubing, having in eachend a small chain with which tension is easily regulated; these are hooked into the eyes above described that represent the origin and insertion of the muscle. The natural movements of muscles are beautifully illustrated and the traction is sufficient, unless structural changes have taken place, to overcome the rigidity of the opposing group; the foot is not only restored to normal position, but is retained and made to go through the natural movements. The treatment of clubfoot by open incision has been warmly advocated by some; I have never seen very encouraging results from the operation.

Tenotomy. Since the introduction of subcutaneous tenotomy in 1834 there has been in all deformities of the foot an indiscriminate cutting of tendons, and only within the past few years has there been a halt in this simple and too common procedure. To the credit of the father of orthopædic surgery in America is to be placed the following rule, which serves as an indication for tenotomy when the conditions are found.

Placing on the stretch the shortened tissues, and applying point pressure; if from this pressure spasmodic contractions ensue, this is proof positive that these tissues have undergone structural changes, that they will not admit of elastic tension; that tenotomy is indicated for the restoration of any deformity that may exist; that these tendons or tissues are contractured and not contracted, and that tenotomy is never indicated, unless structural changes exist.

I am disposed to think, however, that a very small per cent of tendons are found contractured; from a personal examination of several hundred cases, and from the report of my associates at the hospital, very few cases, indeed, were found to present the characteristic symptoms described, but that such conditions and symptoms do exist, there can be no reasonable doubt.

*Bibliography:* Knight's Orthopædia; Robert's Club-foot; Churchill, Face and Foot deformities; Reeves, Bodily deformities; Sayres, Orthopædic Surgery; Prince, Plastic and Orthopædic Surgery.

